# Characterizing Obesity Interventions and Treatment for Children and Youths During 1991–2018

**DOI:** 10.3390/ijerph16214227

**Published:** 2019-10-31

**Authors:** Bach Xuan Tran, Son Nghiem, Clifford Afoakwah, Carl A. Latkin, Giang Hai Ha, Thao Phuong Nguyen, Linh Phuong Doan, Hai Quang Pham, Cyrus S.H. Ho, Roger C.M. Ho

**Affiliations:** 1 Institute for Preventive Medicine and Public Health, Hanoi Medical University, Hanoi 100000, Vietnam; 2 Bloomberg School of Public Health, Johns Hopkins University, Baltimore, MD 21205, USA; carl.latkin@jhu.edu; 3 Centre for Applied Health Economics (CAHE), Griffith University, Brisbane, QLD 4222, Australia; s.nghiem@griffith.edu.au (S.N.); c.afoakwah@griffith.edu.au (C.A.); 4 Institute for Global Health Innovations, Duy Tan University, Da Nang 550000, Vietnam; giang.ighi@gmail.com (G.H.H.); qhai.ighi@gmail.com (H.Q.P.); 5 Center of Excellence in Evidence-based Medicine, Nguyen Tat Thanh University, Ho Chi Minh City 700000, Vietnam; thao.coentt@gmail.com; 6 Center of Excellence in Pharmacoeconomics and Management, Nguyen Tat Thanh University, Ho Chi Minh City 700000, Vietnam; linh91.coentt@gmail.com; 7 Department of Psychological Medicine, National University Hospital, Singapore 119074, Singapore; cyrushosh@gmail.com; 8 Center of Excellence in Behavioral Medicine, Nguyen Tat Thanh University, Ho Chi Minh City 700000, Vietnam; pcmrhcm@nus.edu.sg; 9 Department of Psychological Medicine, Yong Loo Lin School of Medicine, National University of Singapore, Singapore 119228, Singapore; 10 Institute for Health Innovation and Technology (iHealthtech), National University of Singapore, Singapore 119228, Singapore

**Keywords:** scientometrics, obesity, interventions, children, youths, pediatrics

## Abstract

Overweight and obesity have become a serious health problem globally due to its significant role in increased morbidity and mortality. The treatments for this health issue are various such as lifestyle modifications, pharmacological therapies, and surgery. However, little is known about the productivity, workflow, topics, and landscape research of all the papers mentioning the intervention and treatment for children with obesity. A total of 20,925 publications from the Web of Science database mentioning interventions and treatment in reducing the burden of childhood overweight and obesity on physical health, mental health, and society published in the period from 1991 to 2018 were in the analysis. We used Latent Dirichlet Allocation (LDA) for identifying the topics and a dendrogram for research disciplines. We found that the number of papers related to multilevel interventions such as family-based, school-based, and community-based is increasing. The number of papers mentioning interventions aimed at children and adolescents with overweight or obesity is not high in poor-resource settings or countries compared to the growth in the prevalence of overweight and obesity among youth due to cultural concepts or nutrition transition. Therefore, there is a need for support from developed countries to control the rising rates of overweight and obesity.

## 1. Introduction

Obesity is defined as the “abnormal or excessive fat accumulation” that may seriously have an impact on health [1] and is considered one of the most significant health challenges of the 21st century. There are several definitions used in research regarding childhood obesity [2,3,4], yet, none of them are ideal for all studies and the use of definition is based on “practical aspects” [5]. 

Globally, the prevalence of overweight and obesity in children and youth continues to rise. According to World Health Organization (WHO) data, in 2016, nearly one in five children and adolescents between the ages of 5–19 were affected by overweight or obesity [6]. Furthermore, 38.3 million children under the age of five were estimated to be overweight in 2017 [2]. Several low and middle-income settings are leading at this rate, such as Egypt, Fiji, Jordan, Lebanon, and Nicaragua [6,7]. Notably, there was a 48% increase in the prevalence of children and adolescents with overweight and obesity from 2010–2016 in the South-East Asia region alone [8]. 

Childhood overweight and obesity is not only associated with immediate health risks but can progress into adulthood, leading to the development of a host of non-communicable diseases [9,10,11,12,13], or mental health illness [14,15]. Children or adolescents with overweight or obesity are also more likely to be bullied at school [16]. Moreover, overweight and obesity can have deleterious consequences in the later life of children and adolescents. Stigmatization in the workplace [17] increases the chance of being unemployed and having lower income among women with obesity and overweight [18,19]. 

Another matter is the economic impact of childhood overweight and obesity. In the Republic of Ireland, the annual healthcare costs amongst children and adolescents with overweight and obesity were on average €1,709,703 [20]. Research from Australia suggests that the cost to the government as a consequence of children affected by overweight and obesity between the ages of 6–13 is over AUD $43 million per annum [21]. Moreover, even more staggering is the annual direct health cost of childhood overweight and obesity in the USA, which has been estimated to be USD $14 billion [22].

Tackling childhood overweight and obesity requires a multifaceted approach. The WHO proposes that prevention should start before birth, emphasizing the importance of healthy maternal nutrition and gestational weight gain [8,23]. The adage “breast is best” is also true in the prevention of childhood obesity, while also providing the best nutrients for infants during this stage of their life [24]. However, food environments also play an integral role in childhood overweight and obesity, and the WHO has previously recommended policy actions to promote restrictions on the marketing strategies that food and beverage industries target at children. These include, for example, preventing the marketing of foods high in saturated and trans-fats, sugar and salt in any form, in places where children gather (e.g., local sports grounds); internalizing positive emotional food memories being used to develop a healthy lifestyle [25], and ensuring that schools promote physical activity and provide health education [26,27]. 

Treatment targeted towards individuals is also recommended. Diet therapy, for example, can include the introduction of low-fat diets, calorie-controlled diets, or meal replacements. A previously published systematic review has evidenced that there is some merit behind the effectiveness of diet therapy [28]. Physical activity is also crucial for the prevention of childhood overweight and obesity and maintaining healthy body weight. Moreover, there is strong evidence to support the impacts of regular physical activity for the reduction of a risk factor for CVD and diabetes [29,30]. The family and the food environment that a child grows up around can also contribute to promoting weight loss and sustained weight maintenance for children [31,32]. 

Reviews of childhood overweight and obesity literature can help summarize evidence and guide policy development for addressing this global epidemic. Previously, several systematic reviews and meta-analyses in the field of treatment and intervention of obesity for children and adolescents have been conducted [28,33]. These studies provided insights on a defined research question by synthesizing evidence from previous research. However, a limitation of this approach is that these reviews tend to emphasize a unidimensional topic, which makes it difficult to compare with other research domains over time. 

Previously, researchers have applied scientometric analytic approaches to literature reviewing. This method generates a profile of publications for an area of research, and describes the number of publications, citations, downloads, type of journals where this research is published, and patterns of co-authorship, to understand the growth in research productivity and trends specific to that area of research [34,35]. However, scientometric analyses lack essential implications for clinical research, health services improvement, and community interventions, as they do not aim to understand the landscape of topics being researched. Thus, this study aims to describe the growth of research publications regarding interventions for childhood overweight and obesity and to understand the current research landscape. By combining scientometric and content analysis approaches, the authors categorize interdisciplinary topics and interests of the research community regarding interventions for overweight and obesity among children, that can be used to drive future policies.

## 2. Materials and Methods 

### 2.1. Search Strategy and Eligibility

We used the online database Web of Science (WOS) to identify research papers regarding interventions to treat children and adolescents with obesity. The search was conducted in June 2019 and included articles published between January 1991 and December 2018. The WOS database was selected because it is suitable for our objectives, including (1) 254 various disciplines [36], (2) Higher impact journals [37]. Moreover, WOS allows us to download a large amount of data with important information for our analysis, such as the number of times the papers were downloaded and research areas which were assigned by WOS. 

The WOS database search, using keywords, was divided into two steps. Specialists in the field of nutrition and adolescent health found keywords related to “obesity” and “children”. In step 1, they were used to collect research articles and reviews from WOS. The database search process is shown in Table S1. These papers were then downloaded under the .txt format and imported into STATA (version 14.0, STATA Corp., TX, the USA). Using a STATA syntax, we searched specifically for papers that included the terms “trial*”, and “intervention*” in the title or abstract (step 2). 

Papers written in languages other than English were excluded. Additionally, papers other than original research articles or reviews (e.g., editorial, book chapter, letters to the editor, or conference proceedings), and papers where the authors were anonymous or unlisted were also excluded from the current study (Figure S1). 

### 2.2. Data Extraction

Two independent researchers extracted the following data from the final library of papers: (1) author names, (2) publication title, (3) publication year, (4) journal’s title of publication, (5) authors’ keywords, (6) author affiliations, (7) corresponding author details, (8) number of citations, (9) research areas and (10) abstract. Any inconsistencies in the extracted data by the two researchers were resolved by discussion. 

### 2.3. Data Analysis

Data extracted by the independent researchers were transferred into STATA (version 14.0, STATA Corp., TX, the USA). Descriptive statistics were used to generate results for the following publication characteristics: (1) the number of publications produced per year, (2) the number of publications per country (from 1991–2018), (3) the average number of citations for all published papers per year, (4) the frequency of publication downloads in the last six months of 2018 and in the period of 2014–2018, and (5) the average number of publication downloads in the last six months of 2018 and during the period of 2014–2018. Latent Dirichlet Allocation (LDA) was chosen as the method of classifying papers into comparable topics [38,39,40,41,42]. A STATA syntax was used to assign papers to 10 major topics. The data were exported into Microsoft Excel for reading titles and abstracts. Two researchers independently reviewed the titles and abstracts of most cited papers within each research topic to assign the labels correctly for each topic. After that, an expert would discuss with two researchers to unify the name of topics. Besides providing the total number and percentage of research studies by each topic, we ranked the research interests of these research topics based on the total number of its publications in the past five years. Table 1 identifies how the inputs and outputs of each of these analytical methods.

## 3. Results

**Figure 1:**
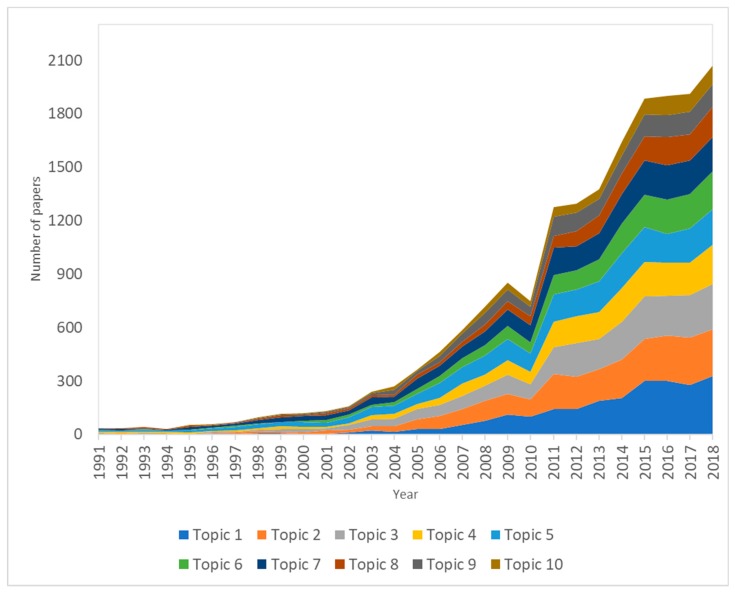
Changes in research topics development in obesity interventions among children and youths. Note: Topic 1: Communities interventions; Topic 2: Pharmacotherapeutic interventions; Topic 3: Epidemiological studies; Topic 4: Family-based, multidisciplinary interventions; Topic 5: Treatment of obesity-related diseases; Topic 6: Psychological and social impairments related to obesity; Topic 7: Treatment of chronic conditions with complications by obesity; Topic 8: Biomedical and preclinical research of obesity-related issues; Topic 9: Community interventions to promote physical activity; Topic 10: Dietary interventions. Topic 1, Topic 2, and Topic 3 received the highest number of papers; meanwhile, Topic 10 had the lowest number of publications.

Table 2 shows the general characteristics of research publications. Between the years 1991 to 2018, there were 20,925 papers related to interventions and treatments for children and adolescents with overweight or obesity. On average, the number of publications each year increased. There was a notable jump in the number of publications between the years 2010 and 2011; 852 and 1,432 publications respectively (roughly a 68% increase in 2011). This phenomenon may be explained by the impact of the “Let’s Move!” campaign [43] and the Affordable Care Act (ACA) on childhood obesity [44]. The total number of usages and the mean use rate of the last five years of papers published in 2013 were the highest compared with that of other years, which shows the recent interest of readers, was significantly higher within the past five years (Figure 1).

Table 3 presents the top 60 countries where the research was conducted. Two-thirds of the studies were conducted in the USA with 1394 papers (20.61%). In the top 10 countries, there were three Asian countries, India, China, and Oman, each of which contributed 4.9%, 3.4%, and 2.9% of the publications, respectively. In Africa, a high percentage of children with apparently healthy BMI-for-age have excessive body fatness [45], such as South Africa (9.4%–school–age children and adolescents 5–19 years, 2016), and Ghana (15%–school children 9–15 years, 2012)

The top ten research topics are presented in Table 4. Topics with the highest volumes of publications included Topic 2: Pharmacotherapeutic interventions on obesity and complications (n = 2475 publications); Topic 1: Community Interventions among Children and Youths (n = 2371 publications); and Topic 3: Epidemiological studies of obesity among children and youth (n = 2350 publications).

Figure 1 depicts the changes in publication volume related to the top ten research topics. In the last five years, Topic 1: School, Community, and System Approaches to Obesity Interventions among Children and Youths, and Topic 2: Pharmacotherapeutic interventions on obesity and related chronic conditions, have attracted the most attention amongst researchers. Notably, treatment for health issues related to obesity (Topic 5 or Topic 7) was one of the main concerns in this field of research.

**Figure 2:**
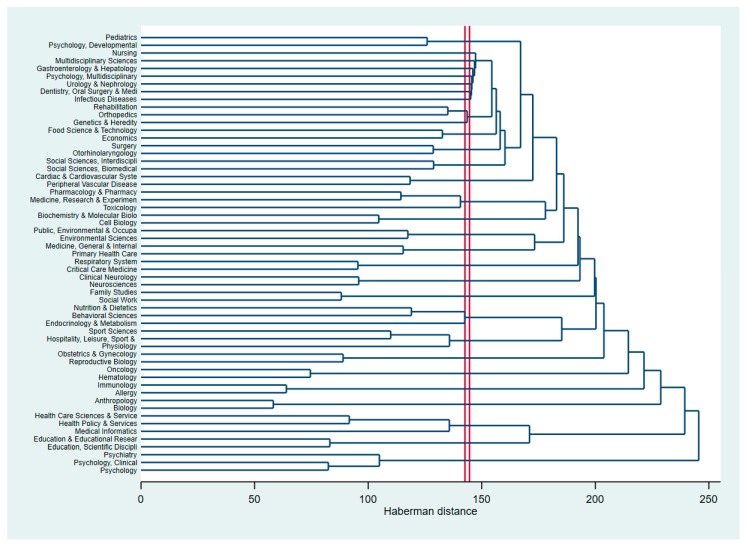
Dendrogram of coincidence of research areas using the WOS classifications.

Figure 2 presents the hierarchical clustering of research disciplines in the intervention and treatment of children and adolescents with obesity. The horizontal axis of the dendrogram shows the distance or dissimilarity between clusters while the vertical represents the research disciplines. Figure 2 shows that research landscapes in the intervention and treatment of children and adolescents with obesity and overweight are rooted in pediatrics and psychology disciplines. These research areas had a close connection with other clinic fields such as Hepatology, Urology, or Dentistry. In the top, we found the integration of (a) Psychiatry, (b) Psychology clinical, and (c) Psychology. This shows a looser connection between Pediatrics and Psychology clinical; meanwhile, associations between obesity and psychiatric disorders has been proved in some studies [46,47]. 

## 4. Discussion

This study aimed to describe the growth of research publications regarding interventions for childhood overweight and obesity and to understand the current research landscape by grouping publications according to similarities in the topic areas being researched. By quantifying the scientometrics profile of publications, it is evident that over time, there has been a gradual increase in the publication of research related to interventions and treatments for children with obesity. Applying the LDA technique, we found that the number of publications related to school and community-based intervention has increased over the period. Moreover, the results highlighted the importance of research support in lower-middle-income countries (LMICs), especially, where the prevalence of childhood obesity has been increased [45].

Unsurprisingly, the majority of the research related to interventions and treatment for childhood obesity was conducted in the United States. India and China were the two lower-middle-income countries that appeared the most in the abstracts (Table 2). However, it should be noticed that the majority of children with overweight and obesity live in lower-setting countries, where the rates are rising faster than in high-income countries [48]. The possible causes of this are various, including the Western diet [49], the low activity level [50], or regarding the cultural perspective, where obesity means beauty, better health, power, and higher socioeconomic status [51]. Each LMIC needs to investigate the causes of this phenomenon due to the difference in the contextual background. Yet, due to the limitation in research funding and facilities, human resources, or communication [52], the contextualized evidence might not be found in the short term. Thus, LMICs might benefit from the active research collaboration with developed countries or countries in the same region, such as India and China. 

By applying our proposed approach, the combination of scientometrics and content analysis, the present study has identified that there has been a wealth of research conducted globally focusing on the development of the pharmacotherapeutic, family-based, school-based, and community-based interventions, especially in the last five years. Meanwhile, seemingly unidimensional approaches, such as diet or nutrition-related interventions, have reduced. This suggests a definite shift in research towards multifaceted intervention and treatment approaches, particularly those that focus on children’s immediate environments. Besides, our studies found that 13.3% of the total papers mentioned pharmacotherapeutic interventions and related chronic conditions. Pharmacotherapy is recommended as a combination method when lifestyle intervention and support from a specialist alone are not enough due to extreme excess weight and related chronic conditions [53]. However, this method should be carefully applied under the supervision of health staff due to some side-effects this may cause to health [54].

The findings suggest some implications for designing interventions, health research, and policy. The number of articles related to the treatment and intervention in poor-resource countries were not high compared with that of high-income countries; meanwhile, the prevalence of obesity and overweight in those settings is increasing. These countries should actively collaborate with other developed countries and countries in the same region (either through their own government or through the support of neighboring/higher-income countries) in order to combat their rising rates of overweight and obesity. Health system strengthening and capacity building, especially in developing countries, will remain as core components of international and national strategies. The developed countries might note the increasing number in the publication regarding the interventions and treatment of childhood obesity. Further research needs to be done to find the most effective strategies of intervention, and treatment of childhood obesity.

Some limitations should be acknowledged. Firstly, the Web of Sciences was the only database used in the analysis. There is a likelihood the low-impact journals, which publish articles from the developing countries, are not being included in the WOS database. Therefore, that may not reflect the development of publications on interventions among children with obesity in developing countries. Future research should consider expanding to other databases to cover the larger number of publications, which contributes more meaningful knowledge to the research field. Secondly, the language of selected papers was restricted to only English. However, there were only 121 papers in other languages, which was a quite small number compared with the total number of papers associated with overweight and obesity in children and youth. Finally, only titles and abstracts were used in the content analysis. However, by quantifying different layers of information (documents by years, citation, and countries where studies conducted) and applying an advanced analytic technique, the Latent Dirichlet Allocation for titles and abstracts analysis [55], we could discover the trend and hidden themes of the research studies [56].

## 5. Conclusions

Research regarding interventions and treatment for children and adolescents with overweight or obesity is still of great interest to researchers over the world. By applying LDA, we identified ten major topics research related to the intervention and treatment of overweight and obesity among children and adolescents. Notably, is the development in the number of publications that mentioned family-based, school-based, and community-based. Besides, the active collaboration from the LMICs’ side, and the support as well as further research on treatments and intervention from developed countries, are needed to reduce the increasing prevalence of childhood obesity.

## Figures and Tables

**Table 1:** Type of data, methods and the results.

Type of Data	Unit of Analysis	Analytical Methods	Presentations of Results
Keywords, Countries	Words	Frequency of co-occurrence	(1) Map of keyword clusters
Abstracts	Papers	Latent Dirichlet Allocation	(2) 10 classifications of research topics
Web of Science (WOS) classification of research areas	WOS research areas	Frequency of co-occurrence	(3) Dendrogram of research disciplines (WOS classification)

**Table 2:** General characteristics of publications.

Year Published	Total Number of Papers	Total Citations	Mean Cite Rate Per Year	Total Usage * Last 6 Months	Total Usage * Last 5 Years	Mean Use Rate ** Last 6 Months	Mean Use Rate ** Last 5 Years
2018	2333	2032	0.87	7750	12,590	3.32	1.08
2017	2147	8532	1.99	3507	20,822	1.63	1.94
2016	2147	15,174	2.36	2704	30,954	1.26	2.88
2015	2103	28,147	3.35	2363	36,240	1.12	3.45
2014	1838	29,916	3.26	1597	33,525	0.87	3.65
2013	1560	36,230	3.87	1140	35,573	0.73	4.56
2012	1459	38,440	3.76	976	27,891	0.67	3.82
2011	1432	42,896	3.74	998	22,267	0.70	3.11
2010	852	33,522	4.37	508	11,302	0.60	2.65
2009	963	39,974	4.15	541	11,023	0.56	2.29
2008	812	39,424	4.41	425	9002	0.52	2.22
2007	668	38,459	4.80	323	8008	0.48	2.40
2006	529	35,718	5.19	284	7142	0.54	2.70
2005	415	32,184	5.54	188	4187	0.45	2.02
2004	307	22,925	4.98	126	3368	0.41	2.19
2003	254	21,127	5.20	99	2187	0.39	1.72
2002	187	16,138	5.08	97	1778	0.52	1.90
2001	151	14,033	5.16	82	2160	0.54	2.86
2000	141	10,951	4.09	96	1610	0.68	2.28
1999	130	11,093	4.27	72	1226	0.55	1.89
1998	117	10,223	4.16	51	1155	0.44	1.97
1997	87	4801	2.51	13	448	0.15	1.03
1996	65	3886	2.60	10	336	0.15	1.03
1995	60	4438	3.08	13	315	0.22	1.05
1994	36	2212	2.46	11	153	0.31	0.85
1993	51	2475	1.87	10	167	0.20	0.65
1992	43	1639	1.41	10	113	0.23	0.53
1991	38	1634	1.54	10	104	0.26	0.55

* Total usage: Total number of downloads. ** Mean use rate: Total number of downloads/Total number of papers.

**Table 3:** Number of papers by countries as study settings.

Rank	Country Settings	Frequency	%	Rank	Country Settings	Frequency	%
1	United States	1394	20.6%	31	Poland	41	0.6%
2	Australia	672	9.9%	32	Chile	40	0.6%
3	India	329	4.9%	33	Malaysia	39	0.6%
4	United Kingdom	268	4.0%	34	Israel	38	0.6%
5	China	230	3.4%	35	Saudi Arabia	37	0.5%
6	Canada	222	3.3%	36	Thailand	36	0.5%
7	Ireland	204	3.0%	37	Switzerland	34	0.5%
8	Oman	197	2.9%	38	Singapore	33	0.5%
9	Brazil	177	2.6%	39	Indonesia	30	0.4%
10	Germany	140	2.1%	40	Wallis and Futuna	30	0.4%
11	Japan	131	1.9%	41	Colombia	29	0.4%
12	Mexico	128	1.9%	42	Viet Nam	29	0.4%
13	Iran	125	1.8%	43	Portugal	28	0.4%
14	New Zealand	125	1.8%	44	Bangladesh	27	0.4%
15	Spain	117	1.7%	45	Niger	25	0.4%
16	Netherlands	116	1.7%	46	Austria	23	0.3%
17	South Africa	107	1.6%	47	Georgia	23	0.3%
18	Sweden	88	1.3%	48	Kuwait	23	0.3%
19	Italy	86	1.3%	49	Nigeria	23	0.3%
20	Taiwan	66	1.0%	50	Argentina	22	0.3%
21	Greece	63	0.9%	51	Egypt	22	0.3%
22	Belgium	62	0.9%	52	Cuba	21	0.3%
23	France	62	0.9%	53	Czech	20	0.3%
24	Denmark	53	0.8%	54	Hungary	20	0.3%
25	Norway	53	0.8%	55	Bulgaria	18	0.3%
26	Turkey	46	0.7%	56	Jersey	18	0.3%
27	Finland	44	0.6%	57	Estonia	17	0.3%
28	Pakistan	44	0.6%	58	Guatemala	17	0.3%
29	Peru	43	0.6%	59	Lebanon	17	0.3%
30	Hong Kong	42	0.6%	60	Ghana	16	0.2%

**Table 4:** The top ten research topics classified using the Latent Dirichlet Allocation method.

Rank by the Highest Volume	Research Topics	n	Percent
Topic 2	Pharmacotherapeutic interventions on obesity and complications	2475	13.30%
Topic 1	Community Interventions among Children and Youths	2371	12.80%
Topic 3	Epidemiological studies of obesity among children and youths	2350	12.70%
Topic 5	Treatment of obesity-related diseases	2296	12.40%
Topic 7	Treatment of chronic conditions with complications by obesity	2082	11.20%
Topic 4	Family-based, multidisciplinary interventions	1889	10.20%
Topic 6	Psychological and social impairments related to obesity	1688	9.10%
Topic 8	Biomedical and preclinical research of obesity-related issues	1276	6.90%
Topic 9	Community interventions to promote physical activity	1264	6.80%
Topic 10	Dietary interventions	851	4.60%

## Supplementary Materials

Supplemental Table S1: Search query for Overweight and Obesity in Children and Adolescents, Figure S1: Selection of papers.
