# Supplementary material for: Characterizing Obesity Interventions and Treatment for Children and Youths During 1991–2018
Source: Int J Environ Res Public Health. 2019 Oct 31;16(21):4227. doi: 10.3390/ijerph16214227 (PMC6863014; doi:10.3390/ijerph16214227)
Supplement: Supplementary file 1 [file ijerph-16-04227-s001.zip › Supplemental Table S1.docx]

**Supplemental Table S1.** Search query for Overweight and Obesity in Children and Adolescents

| **No** | **Results** | **Search query** |
| --- | --- | --- |
| #1 | 402,239 | TS=(Overweight or “Over weight” or Preobes* or Pre-obes* or Obes* or overnutrition or "over nutrition" or “excessive fat accumulation” or “fat levels” or “excess body fat” or “weight disorder” or “High body mass index”) |
| #2 | 2,787,642 | TS=(Child* or Pediatric* or Paediatric* or Infant* or newborn* or bab* or Toddler* or pre-adolescen* or preadolescen* or Adolescen* or Youth* or Youngster* or Teen* or Teenager* or teenage or preschooler* or pre-schooler* or pre-school* or preschool* or “Pre-school child*” or “school-aged child*” or schoolchild* or “school age*” or Schoolage* or “nursery school*” or kindergar* or “primary school*” or “secondary school*” or “grade school*” or “elementary school*” or “high school*” or highschool*) |
| #3 | 84,558 | #2 AND #1 |
| #4 | 83,975 | #2 AND #1  Refined by: [excluding] publication years: (2019) |
| #5 | 68,293 | #2 AND #1  Refined by: [excluding] publication years: (2019) and [excluding] document types: (Meeting Abstract or Proceedings Paper or Editorial Material or Letter or Book Chapter or Correction or News Item or Note or Book Review or Book or Early Access or Reprint or Retracted Publication or Correction Addition or Discussion or Biographical Item or Poetry or Retraction) |
| #6 | 64,241 | #2 AND #1  Refined by: [excluding] publication years: (2019) and [excluding] document types: (Meeting Abstract or Proceedings Paper or Editorial Material or Letter or Book Chapter or Correction or News Item or Note or Book Review or Book or Early Access or Reprint or Retracted Publication or Correction Addition or Discussion or Biographical Item or Poetry or Retraction) and [excluding] authors: (Anonymous) |
| #7 | 64,117 | #2 AND #1  Refined by: [excluding] publication years: (2019) and [excluding] document types: (Meeting Abstract or Proceedings Paper or Editorial Material or Letter or Book Chapter or Correction or News Item or Note or Book Review or Book or Early Access or Reprint or Retracted Publication or Correction Addition or Discussion or Biographical Item or Poetry or Retraction) and [excluding] authors: (Anonymous) and [excluding] languages: (Spanish or German or Portuguese or French or Turkish or Korean or Italian or Polish or Russian or Hungarian or Malay or Greek or Croatian or Serbian or Czech or Japanese or Icelandic or Slovenian or Dutch or Romanian or Chinese or Estonian or Norwegian or Afrikaans or Arabic or Galician or Lithuanian or Persian or Welsh) |
