# Supplementary figures and images for: Characterizing Obesity Interventions and Treatment for Children and Youths During 1991–2018
Source: Int J Environ Res Public Health. 2019 Oct 31;16(21):4227. doi: 10.3390/ijerph16214227 (PMC6863014; doi:10.3390/ijerph16214227)

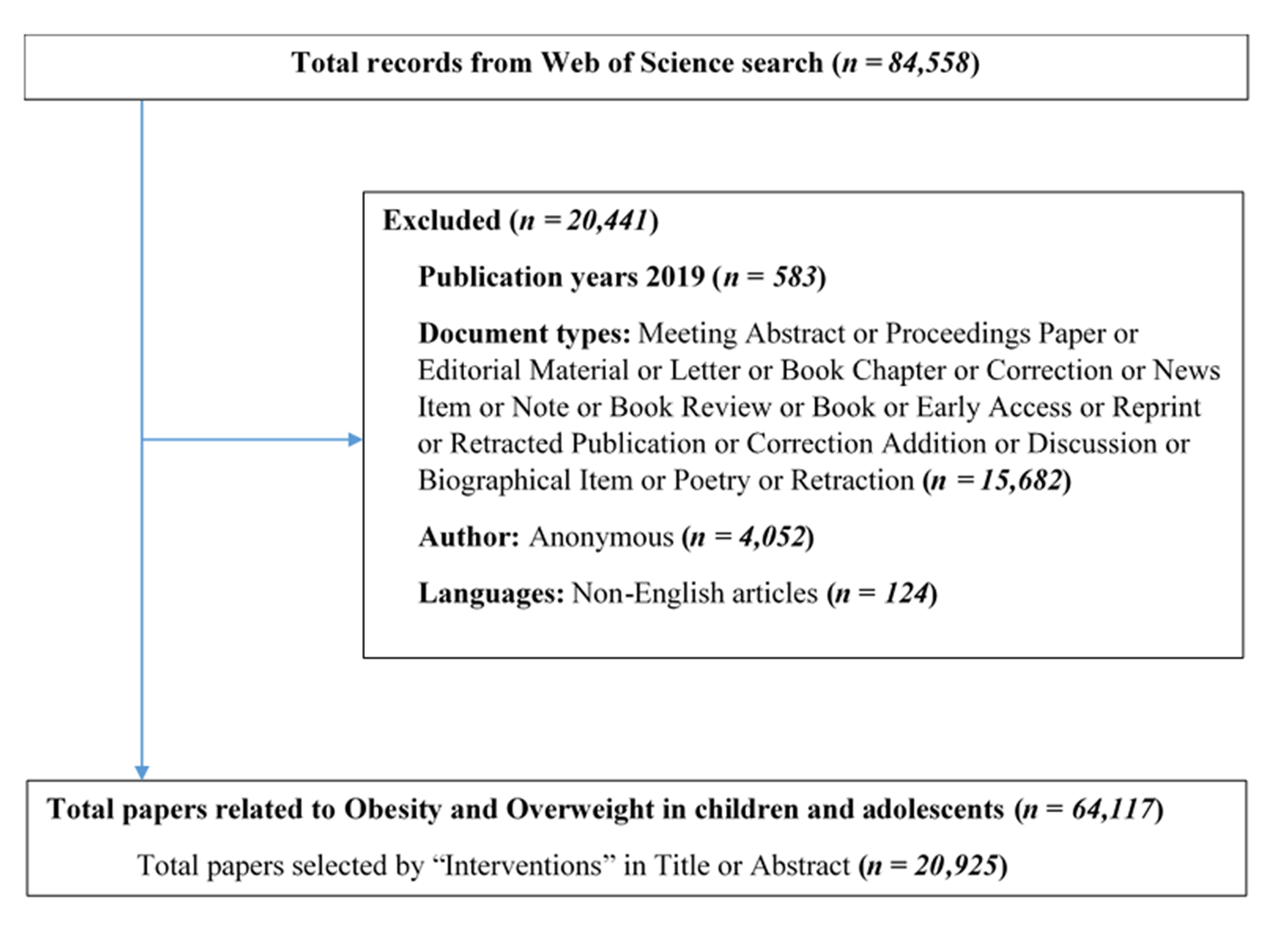

Supplement: Supplementary file 1 [file ijerph-16-04227-s001.zip › Supplemental Figure S1.tif]
